# Diaphragmatic Hernia With Secondary Pneumothorax Due to Intestinal Perforation After Thoracoabdominal Aortic Aneurysm Operation: A Case Report

**DOI:** 10.7759/cureus.61616

**Published:** 2024-06-03

**Authors:** Kazuki Nishimura, Satoshi Hayasaka

**Affiliations:** 1 Anesthesiology and Critical Care, KKR Sapporo Medical Center, Sapporo, JPN

**Keywords:** thoracoabdominal aortic aneurysm surgery, iatrogenic diaphragmatic hernia, intestinal perforation, secondary pneumothorax, diaphragmatic hernia

## Abstract

A male in his 70s with a history of artificial vessel replacement for a thoracoabdominal aneurysm had been treated non-operatively for adhesive bowel obstruction during the past two months. The initial symptom was nausea and the patient was transferred to our hospital because of diffuse abdominal pain. Computed tomography revealed pneumothorax, diaphragmatic hernia, and bowel perforation. A left thoracic drain was inserted and air and clear yellow fluid were drained. Secondary pneumothorax was presumably caused by intestinal perforation associated with diaphragmatic hernia. Although reported cases with secondary pneumothorax associated with diaphragmatic hernia and intestinal perforation are caused by trauma, this complication can occur postoperatively.

## Introduction

While the thoracic cavity is under negative pressure, the abdominal cavity is under positive pressure. Communication between the thoracic and abdominal cavities caused by a diaphragmatic hernia allows the intra-abdominal organs to enter the thoracic cavity. When intestinal perforation occurs in a patient with a diaphragmatic hernia, intra-abdominal free air flows into the thoracic cavity, resulting in secondary pneumothorax [1,2]. We report the case of a patient with a diaphragmatic hernia who developed secondary pneumothorax due to intestinal perforation after a thoracoabdominal aortic aneurysm surgery.

## Case presentation

A male in his 70s had been receiving non-operative treatment for adhesive bowel obstruction for two months. He had a history of acute myocardial infarction (AMI) treated with percutaneous coronary intervention (PCI), chronic kidney disease (CKD), and lumbar spinal canal stenosis. Moreover, one year ago, he had undergone artificial vessel replacement for a thoracoabdominal aneurysm, during which the diaphragm was resected and sutured. Medication included aspirin for coronary disease, nitroglycerin for angina pectoris, furosemide, and bisoprolol fumarate for heart failure.

**Figure 1:**
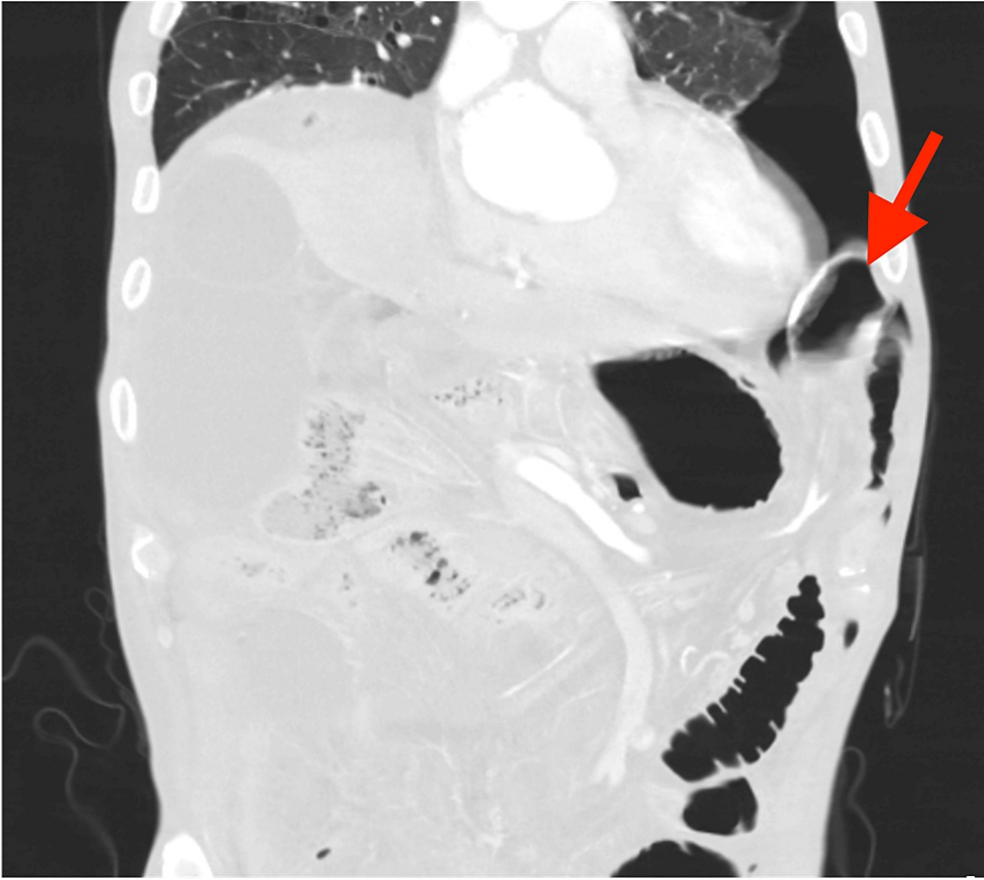
Chest computed tomography showing pneumothorax and diaphragmatic hernia

**Figure 2:**
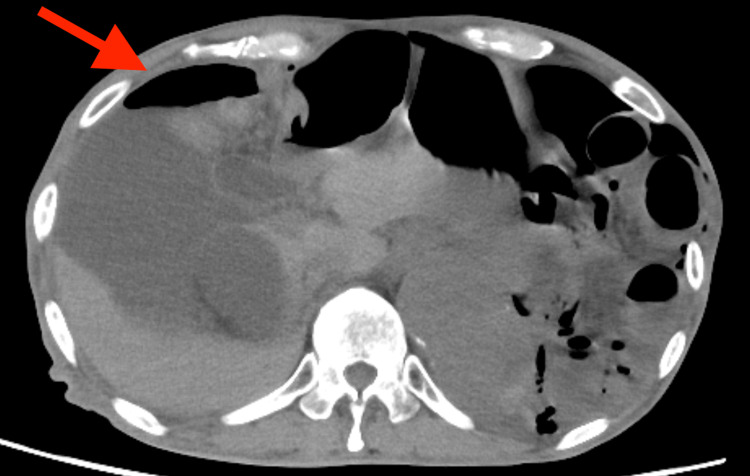
Abdominal computed tomography showing intra-abdominal free air

His initial symptom was nausea and the patient was transferred to our hospital because of diffuse abdominal pain. Upon admission, the patient had a normal level of consciousness, body temperature of 38.8 °C, heart rate of 132 bpm, blood pressure of 100/50 mmHg, respiratory rate of 30 cycles/minutes, and percutaneous oxygen saturation of 96% with 8 L/minute of oxygen administered by a nasal cannula. Clinical examination revealed abdominal distention and peritoneal irritation. Blood tests showed decreased white blood cell count (1000 /μL) and renal dysfunction (blood urea nitrogen 39.1 mg/dL, creatinine 2.15 mg/dL). Thoraco-abdominal CT revealed a diaphragmatic hernia, pneumothorax, and intra-abdominal free air (Figures 1, 2). Therefore, emergency surgery for intestinal perforation was performed.

A secondary cause of pneumothorax was suspected. We speculated that the diaphragmatic hernia caused communication between the thoracic and abdominal cavities and that free air owing to intestinal perforation flowed from the abdominal cavity into the thoracic cavity, resulting in secondary pneumothorax. If this hypothesis were correct, insertion of a thoracic drain would not be necessary to treat pneumothorax. However, if the patient had a spontaneous pneumothorax, he could develop a tension pneumothorax after the induction of anesthesia. After a discussion with the surgeon, we decided to insert a thoracic drain before induction of anesthesia. A left thoracic drain was placed, and air and a clear yellow fluid were drained.

**Figure 3:**
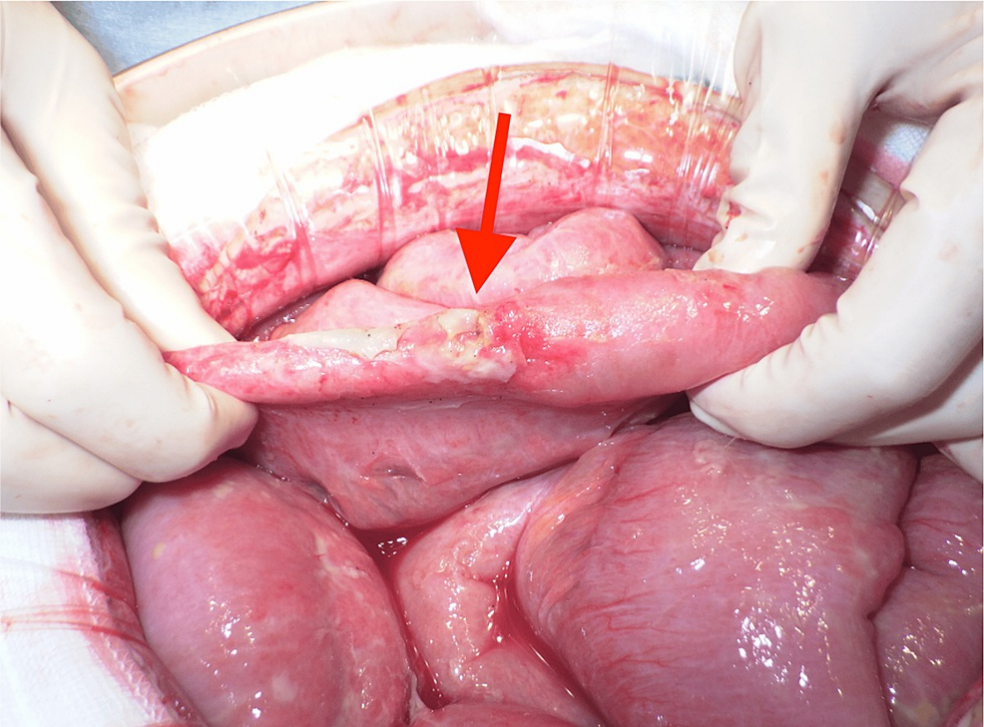
Intraoperative image of the small intestine perforation

During the preparation of the operating room, we placed an A-line and a dialysis catheter (4 lumens), and started antibiotic treatment with carbapenem, while the patient was admitted to the intensive care unit (ICU). We attempted to perform a transthoracic echocardiogram prior to the operation; however, due to pneumothorax, delineation was not successful. We transported the patient to the operating room, verified that the stomach was empty, and performed a rapid sequence introduction with propofol. After induction of anesthesia, noradrenaline at 0.1 mcg/kg/minute was started due to hypotension that could not be managed with phenylephrine. The patient had a myocardial infarction treated with percutaneous coronary intervention, therefore, the cardiac output was expected to be low. Therefore, additional dobutamine administration at 3 mcg/kg/minute was initiated. Due to persistent hypotension, adrenaline at 0.03 mcg/kg/minute and vasopressin at one unit per hour were administered. A 5 cm hernia portal was observed on the lateral aspect of the left diaphragm, and the small intestine was prolapsing into the thoracic cavity, revealing firm adhesions. We decided to abandon the repair of the diaphragmatic hernia during this procedure because of the strong adhesions. The site of small bowel perforation was located several tens of centimeters from the end of the ileum. We removed the perforated site of the small intestine and performed a mechanical anastomosis (Figure 3).

After admission to the ICU, we initiated methylprednisolone (200 mg/day) to address hypotension and possible adrenal insufficiency. Owing to decreased urine output and slightly elevated serum potassium levels, we initiated continuous hemodiafiltration using AN69ST membranes. The following day, blood pressure increased, and continuous adrenaline and vasopressin administration was terminated. The patient was extubated on the third ICU day and discharged on the fourth day when catecholamines were no longer needed. The patient did not develop any infection. On day six of hospitalization, the thoracic drain was removed. After adjustment of diuretic treatment, the patient was discharged on day 17 of hospitalization.

## Discussion

There are three main points to discuss: (i) The patient developed a diaphragmatic hernia after surgery for a thoracoabdominal aortic aneurysm, (ii) Secondary pneumothorax can develop when intestinal perforation occurs in patients with a postoperative diaphragmatic hernia, and (iii) Symptoms could help in establishing the diagnosis of intestinal perforation in patients with diaphragmatic hernia.

First, the patient developed a diaphragmatic hernia after a thoracoabdominal aortic aneurysm operation. Several case reports on postoperative diaphragmatic hernias have described surgical procedures involving organs near the diaphragm, such as the lungs, liver, and esophagus [3-6]. However, we did not find any reports on diaphragmatic hernias after thoracoabdominal aortic aneurysm operation. In the present case, the patient had a history of artificial vascular replacement for a thoracoabdominal aneurysm, during which the diaphragm was resected and sutured. Thus, the diaphragmatic hernia must have been caused by intraoperative manipulations.

Second, a secondary pneumothorax can develop when intestinal perforation occurs in a postoperatively created diaphragmatic hernia. In this case, the diaphragmatic hernia had no sac, allowing free air from the site of intestinal perforation to enter the thoracic cavity through the hernia portal, causing a secondary pneumothorax. Cases of secondary pneumothorax due to posttraumatic diaphragmatic hernia and intestinal perforation have been reported [1,2]. In this case, a similar complication occurred on a postoperative diaphragmatic hernia associated with intestinal perforation.

Third, symptoms can help in establishing the diagnosis of intestinal perforation in patients with diaphragmatic hernia. The diagnosis of intestinal perforation, spontaneous pneumothorax, or tension pneumothorax in patients with diaphragmatic hernia should be based on symptoms and imaging findings. The presence of pneumothorax does not always indicate lung injury, as it may be secondary, associated with an intestinal perforation [7]. Conversely, the presence of intra-abdominal free air does not always indicate intestinal perforation, because free air may originate from a spontaneous pneumothorax [8]. Finally, large amounts of air in the intestinal tract within the thoracic cavity may be misdiagnosed as a tension pneumothorax [9,10].

## Conclusions

We reported a case of diaphragmatic hernia after a thoracoabdominal aortic aneurysm operation, with secondary pneumothorax occurring due to intestinal perforation. Non-operative treatment of bowel obstruction associated with diaphragmatic hernia confers risks of intestinal perforation and secondary pneumothorax. Whenever possible, surgical repair of diaphragmatic hernia should be performed to prevent these complications.
